# High sensitivity C-reactive protein is associated with lower tibial cartilage volume but not lower patella cartilage volume in healthy women at mid-life

**DOI:** 10.1186/ar2380

**Published:** 2008-02-29

**Authors:** Fahad S Hanna, Robin J Bell, Flavia M Cicuttini, Sonia L Davison, Anita E Wluka, Susan R Davis

**Affiliations:** 1 Women's Health Program, Department of Medicine, Central and Eastern Clinical School, Monash University, Alfred Hospital, Prahran, Victoria Australia; 2 The Jean Hailes Foundation, Clayton, Victoria Australia; 3 Department of Epidemiology and Preventive Medicine, Monash University, Prahran, Australia; 4 Baker Heart Research Institute, AMREP Centre, Melbourne, Australia

## Abstract

**Introduction:**

Elevated serum high sensitivity C-reactive protein (hsCRP) has been reported in established osteoarthritis (OA). The aim of this study was to determine whether serum levels of hsCRP are associated with the variation in tibial and patella cartilage volumes in women without evidence of OA.

**Methods:**

Participants were recruited from a database established from the Australian electoral roll, and were aged 40 to 67 years, were not hysterectomized and had no significant knee pain or knee injury in the last 5 years. Tibial and patella cartilage volumes were measured from magnetic resonance imaging (MRI) of each woman's dominant knee and hsCRP measured in serum. Linear regression models were used to explore the major determinants of variation in both tibial and patella cartilage volume and to assess whether serum hsCRP made an independent contribution to variation in the volumes of cartilage in the two knee compartments.

**Results:**

The mean age of the 176 participants was 52.3 ± 6.6 years. Compared with a standard model for tibial cartilage volume that included bone area, age, smoking and alcohol status, the addition of an hsCRP term made an independent negative contribution to variation in tibial cartilage volume, irrespective of whether body mass index (BMI) was included in the model or not. By contrast, using a similar approach, hsCRP did not contribute independently to variation in patella cartilage volume.

**Conclusion:**

In asymptomatic women aged 40 to 67 years, serum hsCRP is independently negatively associated with the volume of tibial but not patella cartilage suggesting that subclinical inflammation may predispose to knee cartilage loss in the tibial compartment. This should be further assessed by a longitudinal study.

## Introduction

Osteoarthritis (OA) is a major cause of morbidity, affecting 60% of men and 70% of women over the age of 65 years [1]. In particular, the prevalence of knee OA has been shown to increase with age throughout the elderly years and affect women more than aged-matched men (11% and 7% respectively) [2]. Progressive destruction of articular cartilage and synovial inflammation are the hallmarks of OA [1], with the severity of clinical disease being proportional to articular cartilage lost [3]. However the pathogenesis of this common condition remains poorly understood. Whether subclinical systemic inflammation precedes the clinical manifestation of OA is not known. Although the systemic inflammatory response that characterizes other forms of arthritis, such as rheumatoid arthritis (RA), is not usually manifest in OA, levels of the acute phase reactant C-reactive protein (CRP) have been found to be elevated in some individuals with established OA [4-6]. However, in established OA, serum levels of CRP have not been found to correlate with either radiographic joint space width in a cross-sectional study [4] or disease severity [5].

Magnetic resonance imaging (MRI) has good tissue contrast and anatomical resolution [7], thus allowing a non-invasive examination of the joint structure in pre-disease or early stage OA. MRI can visualize joint structure directly [8,9] and has been recognized as a valid, accurate and reproducible tool to measure articular cartilage volume [9,10]. Loss of knee cartilage has been shown to be related to knee pain [11] and joint replacement with more than 10% lost before any radiological changes can be detected [12].

As the knee is one of the most common joints to be affected by OA [1], and OA commonly occurs in women in late mid-life, to further explore whether low grade systemic inflammation is associated with characteristics of articular cartilage we have examined the relationships between high sensitivity (hs)CRP and tibial and patella cartilage volumes measured by MRI in asymptomatic women at mid-life. As the evaluation of the direct relationship between hsCRP and knee cartilage is potentially complicated by the concurrent relationships between CRP and increased body fat, notably intra-abdominal fat [13,14], and body mass index and knee cartilage volume [15], we have taken an analytical approach to establish the independent effects of hsCRP on knee cartilage.

## Methods

Participants were women recruited to a cross-sectional study of the role of androgens in women using a database established from the electoral roll in the southern Australian state of Victoria between April 2002 and August 2003 [16]. The database of eligible women was created using the following methodology: women were recruited by telephone using a database of individuals from household addresses selected at random on a weekly basis from Australian electoral areas. Starting addresses were selected at random from the electoral roll for each of the sampling points. Interviews were conducted in person on Saturdays and Sundays between 9.00 am and 4.00 pm. Eight interviews were conducted per sampling point. Only one eligible person was recruited per household, and people recruited to the sample tended to stay on the active database for about 2 years. Women underwent telephone screening and were excluded if they were pregnant or less than 6 weeks post-partum, or had experienced any of the following in the preceding 3 months: an acute psychiatric illness; acute renal, liver, cardiovascular disease or any other acute major illness; gynecological surgery; active malignancy or cancer treatment, excluding non-melanotic skin cancer. All participants provided a detailed medical history in response to specific questioning and those with an active medical condition were not recruited.

Women from the original cross-sectional study were eligible for this study if they were aged 40 to 67 years, had not undergone a hysterectomy and had agreed to be re-contacted for further studies. As our intent was to investigate subjects with no significant current or past knee disease, individuals were excluded if they had had any of the following: a clinical diagnosis of knee OA as defined by American College of Rheumatology criteria, knee pain lasting for > 24 h in the last 5 years, a previous knee injury requiring non-weight bearing treatment for > 24 h or surgery (including arthroscopy), or a history of any form of arthritis diagnosed by a medical practitioner. A further exclusion criterion was a contraindication to MRI including pacemaker, metal sutures, presence of shrapnel or iron filings in the eye, or claustrophobia. Of the 355 women who fulfilled these criteria, 176 remained eligible after further exclusions based on the above or the subject being unlikely to be available to complete the longitudinal protocol of re-assessment at 2 years.

Each participant attended for a single morning fasting blood test and measurement of height (cm) and weight (kg) 1 to 2 years (mean 1.53 years, standard deviation (SD) 0.24 years) prior to their knee joint MRI. At this time each woman completed a questionnaire that provided information about smoking and alcohol consumption. Fasting blood drawn at the time of recruitment to the original cross-sectional study was stored at -80°C until assayed.

The study was approved by the Southern Health Human Research and Ethics Committee and the Monash University Human Research and Ethics Committee, Clayton, Victoria, Australia and all participants gave written informed consent. All participants were identified by number, not by name, and the study was conducted in accordance with the Declaration of Helsinki principles.

### Measurement of hsCRP

Measurement of hsCRP was performed using a particle enhanced immunoturbidimetric assay performed on a Hitachi 917 analyzer (Boehringer Mannheim, Friedrich-Ebert-Str. 10068167 Mannheim, Germany). The assay range was 0.1 to 20 mg/l, with intra-assay coefficients of variation (CVs) of 1.34% at 0.55 mg/l and 0.28% at 12.36 mg/l, inter-assay CVs of 5.7% at 0.52 mg/l and 2.51% at 10.98 mg/l and a detection limit of 0.03 mg/l.

### Measurement of tibial cartilage by MRI

Each woman underwent an MRI scan of the knee of her dominant leg (the one with which she first stepped off) between October 2003 and August 2004. Tibial and patella cartilage volumes were determined by MRI image processing on an independent workstation using the Osiris software as previously described [17,18]. Knees were imaged in the sagittal plane on a 1.5-T whole body magnetic resonance unit (Philips, Eindhoven, The Netherlands) using a commercial transmit-receive extremity coil. The following sequence and parameters were used: a T1-weighted fat-suppressed 3D gradient recall acquisition in the steady state, flip angle 55 degrees, repetition time 58 ms, echo time 12 ms, field of view 16 cm, 60 partitions, 513 × 196 matrix, one acquisition time (11 min 56 s). Sagittal images were obtained at a partition thickness of 1.5 mm and an in-plane resolution of 0.31 × 0.83 mm (512 × 196 pixels). The image data were then transferred to a workstation. The volumes of the medial and lateral tibial cartilage plates and the patella were isolated from the total volume by manually drawing disarticulation contours around the cartilage boundaries on each section. These data were re-sampled by bilinear and cubic interpolation (area of 312 and 312 μm and 1.5 mm thickness, continuous sections) for the final three-dimensional rendering. The volume of the particular cartilage plate was determined by summing the pertinent voxels within the resultant binary volume. A trained researcher read each MRI. The intra-observer CVs for the medial and lateral tibial and patella cartilage volume measures were 3.4%, 2.0% and 2.1%, respectively [17]. A similar method was used to measure patella bone volume [19]. The intra-observer coefficient of variation for patella bone volume measure was 2.2%. Tibial plateau cross-sectional area was used as a measure of tibial bone size. It was directly measured from images reformatted in the axial plane using the Osiris software, as previously described [17]. CVs for the medial and lateral tibial plateau areas were 2.3% and 2.4%, respectively [17]. Body mass index (BMI) was calculated as weight (kg)/height (m^2^).

### Sample size

The number of women eligible for recruitment to this study was limited by the need for their participation in a previous study, being in our desired age range, not having had a hysterectomy and agreeing to be contacted about participation in future studies. The initial number recruited for this study was 176, which gave us a statistical power of 90% to show a correlation as low as 0.25 between the various risk factors and knee cartilage volume (alpha error 0.05, two-sided significance), thus explaining up to 6% of the variance of cartilage volume.

### Statistical analysis

The analytical approach used in this analysis was linear regression with total tibial and patella cartilage volumes as the dependent variables. All variables were continuous except for smoking and alcohol use, which were dichotomous (smoking/non-smoking, drinks alcohol/does not drink any alcohol). The hsCRP data was log transformed to normalize the distribution of data.

For the univariate analysis, F values are provided where F = mean square (regression)/mean square (residual). In the multiple regression analysis ΔF is provided, where ΔF= {sum of squares (regression)(variable in)-sum of squares (regression) (variable out)}/mean square (residual) (variable in).

The r^2 ^value for each linear regression model was used as an indicator of the proportion of variation in the dependent variable explained by the model. The p values provided in the tables 4 and 5 refer to the change (Δ) in the r^2 ^value when a new term is added to the regression model. The variables considered for inclusion in the models were factors known from previous analyses to be important in the determination of cartilage volume (such as bone area/volume [20] and factors known to be associated with serum levels of hsCRP [21]). The order of both adding and removing the natural log (ln) hsCRP and the BMI terms to the linear regression model was deliberately chosen to establish the independent contributions of these factors to the variation in the cartilage volumes. A p value of less than 0.05 was considered to be statistically significant. All analyses were performed using the SPSS statistical package (version 14.0; SPSS Inc., Chicago, IL, USA). Demographic characteristics are presented as mean (SD) or as otherwise specified.

## Results

The mean age of the 176 women included in the analysis was 52.3 (6.6) years. Other participant characteristics are listed in Table 1.

**Table 1:** Characteristics of the study population

Characteristics (*n *= 176)	Mean (SD or %)
Age (years)	52.3 (6.6)
Height (cm)	164 (6.5)
Weight (kg)	72.7(14.1)
Body mass index (kg/m^2^)	27.1 (5.5)
Current smoker	21(11.9%
Currently consumes some alcohol	138 (78.9%)
Total tibial cartilage volume (cm^3^)	3.27 (0.55)
Total patella cartilage volume (cm^3^)	2.54 (0.55)
Median high sensitivity C-reactive protein (mg/l)	1.81 mg/l (range 0 to 43.9)

The univariate results are presented in Tables 2 and 3. For tibial cartilage volume, the variables of particular interest from the univariate analysis included bone area, age and ln hsCRP. For the patella cartilage volume, the variables of interest included bone volume, age, BMI and ln hsCRP.

**Table 2:** Univariate results of tibial cartilage volume (mm^3^)

	F	Significance of F
Bone area	28.81	< 0.001
Age	10.23	0.002
Smoking	1.32	0.25
Alcohol	2.00	0.16
BMI	0.49	0.49
ln hsCRP	12.87	< 0.001

F = the regression mean square divided by the residual mean square. BMI, body mass index; ln hsCRP, natural log high sensitivity C-reactive protein.

**Table 3:** Univariate results of patella cartilage volume (mm^3^)

	F	Significance of F
Bone volume	62.739	< 0.001
Age	5.04	0.026
Smoking	0.52	0.47
Alcohol	1.75	0.19
BMI	7.73	0.006
ln hsCRP	7.04	0.009

F = The regression mean square divided by the residual mean square. BMI, body mass index; ln hsCRP, natural log high sensitivity C-reactive protein.

The r^2 ^values for factors potentially contributing to total tibial cartilage and patella volumes are shown in Tables 4 and 5. For total tibial cartilage, total bone area explained 14% of the variation in volume. The addition of age increased the proportion of the variation in cartilage volume explained to 21%. The further addition of smoking and alcohol to this model did not significantly increase the proportion of variation in total tibial cartilage volume explained, however we retained these variables in the analysis to form our standard model against which to test the addition of both BMI and ln hsCRP. There was no significant additional benefit with the addition of BMI. However, the inclusion of the CRP term increased the proportion of cartilage volume variation explained to 28%: this was a highly statistically significant increase in the r^2 ^value, and this was seen whether BMI was included in the model or not (Table 4).

**Table 4:** Total tibial cartilage volume r^2 ^values and p values for Δr^2 ^for different linear regression models

Model for total tibial cartilage volume	r^2^	p Value for Δ r^2 ^compared with the specified model	ΔF with addition to the model (p value for ΔF is the same as for Δr^2^)
Bone area only (+)	0.14	< 0.001	N/A
Bone area (+) age (-)	0.21	< 0.001 compared with bone area only	14.84
Bone area (+) age (-) smoking, alcohol	0.22	0.43 compared with bone area and age	0.86
Bone area (+) age (-) smoking, alcohol, BMI	0.22	0.53 compared with bone area, age, smoking and alcohol	0.39
Bone area (+) age (-) smoking, alcohol, ln hsCRP (-)	0.28	< 0.001 compared with bone area, age, smoking, alcohol	13.15
Bone area (+) age (-) smoking, alcohol, BMI ln hsCRP(-)	0.28	< 0.001 compared with bone area age smoking alcohol	7.23 (both variables added together); if BMI added first, ΔF = 0.43 (p for ΔF = 0.51) followed by lnCRP ΔF = 13.99, (p for ΔF < 0.001); if ln CRP added first, ΔF = 13.15, (p for ΔF < 0.001) followed by BMI ΔF = 1.28, (p for ΔF = 0.26)

Parenthesis indicate the sign of regression coefficients statistically significant at the 5% level. BMI, body mass index (kg/m^2^); ln hsCRP, natural log high sensitivity C-reactive protein.

**Table 5:** Patella cartilage volume r^2 ^values and p values for Δ r^2 ^for different linear regression models

Model for total patella cartilage volume	r^2^	p Value for delta r^2 ^compared with the specified model	ΔF with addition to the model (p value for ΔF as for Δr^2^)
Bone volume only (+)	0.27	< 0.001	N/A
Bone volume (+) age (-)	0.30	0.003 compared with bone volume only	8.93
Bone volume (+) age (-) smoking, alcohol	0.31	p = 0.65 compared with bone volume and age	0.43
Bone volume (+) age (-) smoking, alcohol, BMI	0.32	p = 0.06 compared with bone volume, age, smoking and alcohol	3.54
Bone area (+) age (-), smoking, alcohol, ln hsCRP	0.32	p = 0.19 compared with bone volume, age, smoking, alcohol	1.78
Bone area (+) age (-) smoking, alcohol, BMI ln hsCRP	0.33	p = 0.15 compared with bone volume age smoking alcohol	1.92 (both variables added together); if BMI added first, ΔF = 3.54, (p for ΔF = 0.06) followed by lnCRP ΔF = 0.31, (p for ΔF = 0.58); if ln CRP added first, ΔF = 1.78, (p for Δ= F 0.19) followed by BMI ΔF = 2.05, (p for ΔF = 0.15).

Parenthesis indicate the sign of regression co-efficients statistically significant at the 5% level. BMI, body mass index (kg/m^2^); ln hsCRP, natural log high sensitivity C-reactive protein.

For the patella, 27% of the variation in patella cartilage volume was associated with patella bone volume. This was increased significantly to 30% with the addition of age but was essentially unchanged by the addition of the smoking and alcohol terms, although again we retained these terms in our standard model against which to test the effect of the addition of the BMI and ln hsCRP terms. Addition of either BMI or ln hsCRP separately increased the proportion of variation in patella cartilage volume explained to 32% (the change did not reach statistical significance at the 5% level in either case). Although the addition of both the ln hsCRP and BMI terms increased the r^2 ^value by 2%, again the change did not reach statistical significance at the 5% level (Table 5).

## Discussion

The principal finding of this study was that in women at mid-life, the level of hsCRP in serum made a significant independent contribution to the variation in the volume of total tibial cartilage measured approximately 12 to 18 months later. This observation was true whether or not the analysis was controlled for BMI. This observation did not hold true for the patella compartment. Although BMI is a major determinant of CRP [21], our analysis indicates that the impact of hsCRP is not purely mediated through BMI.

While increased hsCRP has been reported in the early phases of OA [22], this study provides the first evidence of an independent relationship between hsCRP and tibial cartilage volume in asymptomatic individuals. Although OA is not considered a classical inflammatory arthropathy, it is characterized by intra-articular inflammation manifest as synovitis. In this study we were not able to assess synovial effusion or thickness from the MRI. However, recent studies have shown a significant relationship between hsCRP levels and the degree of synovial inflammatory infiltration in OA [23] and RA [24].

CRP is an acute-phase protein that is produced in large amounts by hepatocytes, upon stimulation by the cytokines interleukin (IL)6, tumor necrosis factor α (TNFα) and IL1, during an acute-phase response. Recent studies consistently indicate that both TNFα and IL1 play a key role in cartilage destruction in OA [25,26]. Although OA disease activity appears to be limited to involved joints, release of cytokines into the systemic circulation during the subclinical phase of OA may explain the association we have seen between hsCRP and tibial cartilage volume. The role of CRP in OA may be complex, possibly involving specific haplotypes of the CRP gene [27]. The relationship between CRP levels and BMI may also be complex and involve modulation of CRP gene expression within adipose tissue [28].

Why the relationship with hsCRP was seen in the tibial compartment but not the patella compartment is unclear. However, we have previously reported a lack of association between patella and tibial cartilage loss over 2 years, suggesting that pathogenetic mechanisms for OA in the patellofemoral and tibiofemoral joint may differ [19]. The assessment of the presence/absence of cartilage defects provides another method of assessing cartilage health. In our study, the independent contribution seen by hsCRP to variation in tibial cartilage volume was not seen between hsCRP and the presence of cartilage defects in the tibiofemoral compartment (data not shown). Our findings that age and bone size influence knee cartilage volume corroborate previous findings [29].

Strengths of our study include the community-based recruitment of asymptomatic women, the relatively large number of study participants, and measurement of CRP by a highly sensitive assay. A potential limitation of this study is that hsCRP was only measured on one occasion. Although serial sampling of CRP would have been optimal, it would not have been feasible as many of the women in rural areas had to travel considerable distances for blood sampling. To limit the possibility that CRP levels may have been affected by intercurrent illness, women were asked not to attend for their blood test on a day they were not well. Another limitation to our study is that the cross-sectional design does not provide information regarding the usefulness of hsCRP in predicting cartilage loss. However, re-assessment of our study population after 2 years will provide data for the predictive value of hsCRP for tibial cartilage loss.

## Conclusion

That hsCRP measured several months prior to MRI scanning of the knee was significantly associated with less tibial cartilage volume suggests the possibility that the presence of subclinical systemic inflammation has a role in the early stages of the disease process that precedes symptomatic OA. However, the present report was a cross-sectional study and none of the women in this study had clinical evidence of OA. Nonetheless, our findings indicate that assessing whether inflammation precedes clinical OA merits specific investigation. If activation of inflammation is found to have a pathogenic role in OA development this could have implications as a target for preventative therapy.

## List of abbreviations

BMI = body mass index; CRP = C-reactive protein; CV = coefficient of variation; hsCRP = high sensitivity CRP; IL = interleukin; ln = natural log; OA = osteoarthritis; RA = rheumatoid arthritis; SD = standard deviation; TNFα = tumor necrosis factor α.

## Competing interests

The authors declare that they have no competing interests.

## Authors' contributions

FH was responsible for data collection and analysis and interpretation of the data. RB was responsible for analysis and interpretation of the data. AW performed data analysis. FC and SDavis were responsible for study design and data analysis and interpretation and SDavison reviewed the final draft. All authors prepared, read and approved the final manuscript.
